# Neuroprotective effect of ranolazine improves behavioral discrepancies in a rat model of scopolamine-induced dementia

**DOI:** 10.3389/fnins.2023.1267675

**Published:** 2024-01-12

**Authors:** Shereen M. Samir, Hend M. Hassan, Rasha Elmowafy, Eman Mohamed ElNashar, Mansour Abdullah Alghamdi, Mona Hmoud AlSheikh, Norah Saeed Al-Zahrani, Faten Mohammed Alasiri, Mona G. Elhadidy

**Affiliations:** ^1^Department of Medical Physiology, Faculty of Medicine, Mansoura University, Mansoura, Egypt; ^2^Department of Human Anatomy and Embryology, Faculty of Medicine, Mansoura University, Mansoura, Egypt; ^3^Department of Medical Biochemistry and Molecular Biology, Faculty of Medicine, Mansoura University, Mansoura, Egypt; ^4^Department of Anatomy, College of Medicine, King Khalid University, Abha, Saudi Arabia; ^5^Genomics and Personalized Medicine Unit, College of Medicine, King Khalid University, Abha, Saudi Arabia; ^6^Department of Physiology, College of Medicine, Imam Abdulrahman Bin Faisal University, Dammam, Saudi Arabia; ^7^Department of Clinical Biochemistry, College of Medicine, King Khalid University, Abha, Saudi Arabia; ^8^Pharmacist in King Fahad Armed Forces Hospital Khamis Mushait, Khamis Mushait, Saudi Arabia; ^9^Department of Medical Physiology, Faculty of Medicine, Al-Baha University, Al-Baha, Saudi Arabia

**Keywords:** ranolazine, scopolamine, dementia, apoptosis, GFAP, tau, rats

## Abstract

**Background:**

Ranolazine (Rn), an antianginal agent, acts in the central nervous system and has been used as a potential treatment agent for pain and epileptic disorders. Alzheimer’s disease (AD) is one of the most prevalent neurodegenerative diseases and the leading factor in dementia in the elderly.

**Aim:**

We examined the impact of Rn on scopolamine (Sco)-induced dementia in rats.

**Methods:**

Thirty-two albino male rats were divided into four groups: control, Rn, Sco, and Rn + Sco.

**Results:**

A significant decrease in the escape latency in the Morris water maze test after pre-treatment with Rn explained better learning and memory in rats. Additionally, Rn significantly upregulated the activities of the antioxidant enzymes in the treated group compared to the Sco group but substantially reduced acetylcholinesterase activity levels in the hippocampus. Moreover, Rn dramatically reduced interleukin-1 β (IL-1β) and IL-6 and upregulated the gene expression of brain-derived neurotrophic factor (BDNF). Furthermore, in the Sco group, the hippocampal tissue’s immunohistochemical reaction of Tau and glial factor activating protein (GFAP) was significantly increased in addition to the upregulation of the Caspase-3 gene expression, which was markedly improved by pre-treatment with Rn. The majority of pyramidal neurons had large vesicular nuclei with prominent nucleoli and appeared to be more or less normal, reflecting the all-beneficial effects of Rn when the hippocampal tissue was examined under a microscope.

**Conclusion:**

Our findings indicated that Rn, through its antioxidative, anti-inflammatory, and anti-apoptotic effects, as well as the control of the expression of GFAP, BDNF, and Tau proteins, has a novel neuroprotective impact against scopolamine-induced dementia in rats.

## Graphical abstract

Ranolazine improves scopolamine-induced hippocampal changes evaluated by the assessment of oxidative stress, pro-inflammatory markers, apoptosis, astrocytes and AChE activities, and Tau protein expression. MDA, malondialdehyde; GSH, reduced glutathione; SOD, superoxide dismutase; AchE, acetylcholine esterase; BDNF, brain-derived neurotrophic factor; IL-1 and IL-6, interleukins 1 and 6.

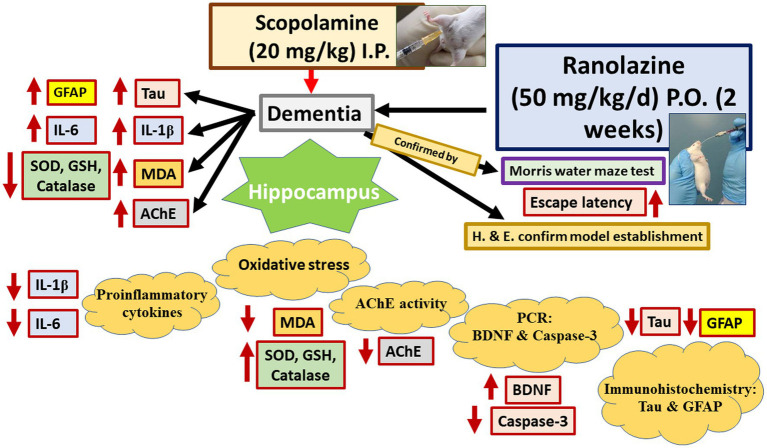

## 1 Introduction

Alzheimer’s disease (AD) is a catastrophic neurodegenerative disorder and a major health problem that is characterized by a decline in cognitive and behavioral abilities as a result of cholinergic nerve system dysfunction (Scarpini et al., 2003; Tiwari et al., 2019). Moreover, Chen et al. (2022) and Hasselmo (2006) reported that patients with AD had brains with impaired cholinergic activity. A number of cholinergic medications have been proven effective in treating or ameliorating AD in trials. These medications work by preventing acetylcholine (ACh) deficiency and raising ACh levels in the brain (Drever et al., 2007; Majdi et al., 2020). Acetylcholinesterase (AChE) inhibitors, such as galantamine, rivastigmine, and donepezil, which momentarily increase the availability of ACh at cholinergic synapses, are the most commonly used category of medications for AD (Lanctôt et al., 2009; Chen et al., 2022). Discomfort, nausea, and hepatotoxicity are just a few of the side effects of the medications that have been licensed for the treatment of AD patients with cognitive impairment (Lahiri et al., 2003; Mitra et al., 2019).

Dementia refers to a syndrome that is characterized by a steady decline of cognitive abilities, which are commonly associated with neuropsychiatric symptoms such as apathy, agitation, and depression. Alzheimer’s disease and cerebrovascular ischemia are the two most common causes of dementia, and no more than 1.5% of mild to moderate cases of dementia are fully reversible (Vijayan and Reddy, 2016).

The tropane alkaloid medication scopolamine (Sco) interferes with cholinergic transmission to generate competitive antagonism at muscarinic acetylcholine receptors (mAChRs), affecting learning and short-term memory in both mice and humans (Iversen, 1997). As a result, the administration of Sco to animals is utilized as an experimental model of memory loss and cognitive decline (Bartus et al., 1982). The central nervous system’s (CNS) expression of cAMP response element-binding protein (CREB) and brain-derived neurotrophic factor (BDNF) is decreased as a result of its administration, which also causes dysregulation of the brain’s cholinergic system and memory circuits. Synaptic plasticity and memory function are governed by BDNF, which is related to CREB activation. CREB is intimately connected to hippocampus learning and memory (Tyler et al., 2002).

The standard treatment for AD is acetylcholinesterase inhibitors. However, they have a variety of undesirable effects, including dizziness, disorientation, nausea, vomiting, and cardiac arrhythmias (Winslow et al., 2011). Consequently, there is a growing need for medicinal treatments that are efficient and safe.

Ranolazine (Rn) is an antianginal agent utilized for the treatment of chronic angina when other medications are unable to sufficiently control it. It is noteworthy that, in clinical studies, Rn does not significantly affect arterial blood pressure or resting heart rate, in contrast to the majority of other cardiovascular drugs, which either cannot be taken at the supposed dose or are discontinued due to such side effects (Kaplan et al., 2022). Rn has been suggested as a treatment for pain and epileptic diseases since it also has CNS effects (Park et al., 2013; Peters et al., 2013). Numerous studies have assessed Rn′s effects on the central nervous system under the belief that it might function as a neuroprotective medication. In a study, Rn was incubated for 24 h with primary cultures of astrocytes and neurons, and the therapy boosted cell survival and proliferation. In addition, it decreased the expression of the pro-inflammatory cytokines interleukin-1 and tumor necrosis factor but increased the expression of the anti-inflammatory PPAR protein (Aldasoro et al., 2016).

Since the effect of Rn on the brain’s cognitive and behavioral functions has not been studied previously, this research study aimed to assess the potential neuroprotective effect of Rn against Sco-induced dementia in male rats.

## 2 Materials and methods

### 2.1 Chemicals used

Ranolazine and scopolamine hydrobromide were obtained from Sigma Chemical Co. (St. Louis, MO, United States).

### 2.2 Ethical approval

This study was approved by the Mansoura University-Animal Care and Use Committee (MU-ACUC) in accordance with NIH and EU standards for animal care (Code: MED.R.23.06.17). The rats were kept in veterinary-supervised housing at Mansoura University’s Faculty of Veterinary Medicine, where the experiment was conducted. The use of rats and the suffering of the animals were kept to a minimum.

### 2.3 Experimental animals

A total of 32 adult male albino rats weighing 180–220 g and aged 3–4 months old were used in the investigation. In each of the metal cages, four rats were maintained under a veterinarian’s care. They were kept at a constant temperature of 20°C, at a humidity level of 50%, and under 12/12 h dark/light environments. They were free to get water and an ordinary diet.

### 2.4 Experimental design

After 2 weeks of acclimatization, the rats were randomly allocated into 4 groups of 8 rats each; rats in the control group received 0.9% normal saline solution orally via gastric gavage; rats in the Rn-treated group were treated with 50 mg/kg/day of Rn that was given orally via gastric gavage for 2 weeks (Wang et al., 2019); rats in the Sco-induced dementia group were injected intraperitoneally with 20 mg/kg scopolamine hydrobromide dissolved in saline (Zaki et al., 2014); and rats in the scopolamine-induced dementia group was pre-treated with 50 mg/kg/day of Rn for 2 weeks.

### 2.5 Behavioral assessment

The Morris water maze test was performed 30 min following the Sco injection. Phases of learning (acquisition) and retention were included in the test. The maze is made up of a circular pool that is divided into four equal quadrants and is 150 cm in diameter and 45 cm in height and 45 cm water height as well. A circular platform with a diameter of 4.5 cm was positioned in one pool quadrant 1 cm below the water surface to facilitate memory learning. Each rat underwent four consecutive trials, separated by 5 min. The rats were slowly dropped into the pool water between quadrants that faced the pool’s wall. Then, each rat had 120 s to find the platform. Rats were guided to the platform and given permission to stay there for 20 s if they were unable to reach it within 120 s. A timer was used to detect how long it took for each rat to get to the platform (escape latency). The platform was taken down and the entry latency to the platform quadrant was measured for memory retention (4 h after the previous learning session) (Hassan et al., 2022).

### 2.6 Specimen collection

Immediately after performing behavioral tests, rats were euthanized by giving an overdose of sodium thiopental (1 mL, intraperitoneal), and their hippocampi were rapidly dissected out. One of the two hippocampi was preserved in 10% formalin and kept for histopathological examination. The other hippocampi were divided into two parts; the first one was homogenized in ice-cold 50 mM phosphate buffer (pH 7.4) to prepare 10% homogenate. For 20 min, the homogenates were centrifuged at 10,000 rpm to remove any cell debris, unbroken cells, erythrocytes, nuclei, and mitochondria. The supernatant was used for the estimation of oxidative stress markers, IL-1, IL-6, BDNF, and AChE activity. The other part was kept fresh in an RNA solution for real-time PCR.

### 2.7 Biochemical studies

#### 2.7.1 Estimation of oxidative stress markers levels in hippocampal homogenates

The levels of malondialdehyde (MDA) (Zamora Rodríguez et al., 2007), catalase (Aebi, 1984), reduced glutathione (GSH) (Beutler et al., 1963), and superoxide dismutase (SOD) (Kakkar et al., 1984) were detected in hippocampal homogenates using commercial colorimetric kits purchased from a biodiagnostic company from Cairo, Egypt. Based on the colorimetric principle, the absorbance was measured spectrophotometrically at specific wavelengths following the manufacturer’s instructions.

#### 2.7.2 Assessment of pro-inflammatory cytokines (IL-1β and IL-6) levels in the hippocampal homogenates

The levels of both IL-1 β and IL-6 were measured in accordance with the protocol of Hsu et al. (2005) using both rat IL-6 ELISA kit (ERA32RB) and rat IL-1β ELISA kit (ERIL1B) from Invitrogen, Thermo-Fisher Scientific, United States. Briefly and according to the manufacturer’s protocol, 100 μL of diluted samples were added to the wells and incubated for 2.5 h at room temperature. The solution was discarded and washed four times with 1X wash buffer (300 μL). Subsequently, 100 μL of the biotin conjugate was added to each well. After incubation and washing, 100 μL of Streptavidin-HRP solution was added and washed after 45 min. Then, 100 μL of TMB substrate and 50 μL of stop solution were added to each well. The plate was read at 450 nm.

#### 2.7.3 Detection of acetylcholinesterase activity in the hippocampal homogenates

According to the procedure described by Ellman et al. (1961) and Gorun et al. (1978), the activity of acetylcholinesterase was assessed in the hippocampal homogenate using a Rat AChE (acetylcholinesterase) ELISA kit (Elabscience, United States). Briefly and according to the manufacturer’s protocol, 100 μL of samples were added to the wells and incubated for 90 min at 37°C. The solution was discarded and 100 μL Biotinylated Detection Ab working solution was added to each well immediately. After incubation for 60 min at 37°C, the plate was washed three times. Subsequently, 100 μL of Streptavidin-HRP working solution was added and washed after 30 min. Then, 90 μL of the substrate reagent and 50 μL of stop solution were added to each well. The plate was read at 450 nm.

### 2.8 Quantification of BDNF and caspase-3 mRNA by real-time reverse transcription-PCR

RNA triazole (Invitrogen, Carlsbad, CA, United States) was used to extract total RNA from the hippocampus in accordance with the manufacturer’s recommendations. A Nanodrop ND-1000 spectrophotometer was used to measure RNA concentrations and purities. Using the manufacturer’s instructions, the RNA PCR kit (TaKaRa, DaLian, China) was used to create complementary DNA (cDNA) from total RNA. For the qPCR analysis, a Trans Start Top Green qPCR Super Mix kit was used (40 cycles of 95°C for 30 s, 55°C for 30 s, and 72°C for 30 s, starting with initial template denaturation at 95°C for 5 s). Using the Q-Gene program, the normalized expression of BDNF and Caspase-3 against β-actin was calculated using the cycle threshold values. The primer pairs sequences were as follows: β-actin, F: 5’ TAGTTGCGTTACACCCTTTCTTG-3′, R: 5’-TCACCTTCACCGTTCCAGTT- 3′; BDNF, F: 5′-GAAGCTCAACCGAAGAGCTAAA-3′, R: 5′-AGCCTTCATGCAACCGAAGTA-3′; and Caspase-3, F: 5’-AGCTTCTTCAGAGGCGA, R: 5’-GGACACAATACACGGGATCT-3′. The internal reference gene β-Actin was used for the normalization of gene expression levels. PCR was analyzed using the ΔΔ cycle threshold method (Livak and Schmittgen, 2001).

### 2.9 Histopathological and immunohistochemical studies

Hippocampal tissues were fixed in 10% phosphate-buffered formaldehyde, embedded in paraffin, and sectioned into 5 μm sections. Hematoxylin and eosin were used to stain the sections. In immunohistochemistry, endogenous peroxidases were suppressed at 0.03% H_2_O_2_. The antigen was then blocked with 5% bovine serum albumin in tris-buffered saline following a 20 min microwave antigen retrieval procedure with a pH-neutral sodium citrate buffer. GFAP (ab7260, 1:1000 dilution) and tau (ab32057, 1:1000 dilution) primary antibodies were subsequently applied to the sections for an additional overnight duration at 4°C. In accordance with the recommendations of the manufacturer (Sigma-Aldrich, St. Louis, MO, United States), an ABC kit was used to identify the reaction. Following that, sections were dried, mounted using synthetic glue, and counterstained with hematoxylin (Ramos-Vara and Miller, 2014).

### 2.10 Morphometric studies

Five randomly spaced sections from each rat in each group were studied in order to calculate the percentage of positive tau and GFAP immunoreaction in the hippocampal tissue. Using a 40x objective, the area percentage of immunological expression was estimated (area: 0.071 mm^2^). Using the Image-j computerized image analysis system (version 1.48), the immune-positive reaction was studied. Three separate-colored images—green, brown, and blue—were created using the color deconvolution plugin and the H-DAB vector. The DAB images, which were brown in color, were calibrated using area fraction. For greater precision, the threshold was adjusted (Hassan et al., 2022).

### 2.11 Statistical analysis

The data were examined using IBM SPSS for Windows, version 26.0. The distribution of the data was determined using the Shapiro test for normality. Data were provided as mean ± SD. The (0.05) level was used to determine the significance. The one-way ANOVA test was used to compare the means of the 4 research groups, and the post-hoc Tukey’s test was performed to determine pairwise comparisons.

## 3 Results

### 3.1 Morris water maze test results

During the initial training period, there was no significant difference among the study groups regarding the escape latency time (*p* = 0.02).

As described in Table 1, rats in the Sco group demonstrated a significant increase in the mean escape and entry latency times of the rats (*p* ≤ 0.001 of both) as compared to those in the control group rats. However, rats in the Rn + Sco group showed a significant decline in the mean escape and entry latency times of the rats (*p* ≤ 0.001 of both) as compared to those in the Sco group, while no significant difference was detected between the control and Rn groups.

**Table 1:** Morris water maze test results.

	Control	Rn	Sco	Rn + Sco	*p* value
Escape latency (s)	33.57 ± 3.4	26.57 ± 2.5	45 ± 2.2^a^	33.7 ± 2.8^b^	≤ 0.001*
Entry latency (s)	10.57 ± 1.5	10.71 ± 1.9	45.57 ± 2.7^a^	20.57 ± 3.2^b^	≤ 0.001*

a Comparison between the Sco and control groups (*p* value ≤ 0.001).

b Comparison between the Sco + Rn and Sco groups (*p* value ≤ 0.001).*p value ≤ 0.05.

### 3.2 Effect of ranolazine administration on oxidative stress, pro-inflammatory cytokines, and acetylcholinesterase activity in the hippocampal tissue of the study groups

As illustrated in Table 2, rats in the Sco group demonstrated a substantial decrease in the levels of SOD, GSH, and catalase activity (*p* ≤ 0.001 for all) and a significant elevation in the levels of MDA and AChE activity (*p* ≤ 0.001 for both) compared to the control group.

**Table 2:** Effect of scopolamine and ranolazine on the level of antioxidants, pro-inflammatory cytokines, and acetylcholinesterase (AChE) activity in the hippocampal tissue.

	Control	Rn	Sco	Rn + Sco	*p* value
SOD (U/g tissue)	26.3 ± 1	25.9 ± 0.8	12.3 ± 1.4^a^	21.07 ± 0.9^b^	≤ 0.001*
GSH (nmol/g tissue)	9.7 ± 0.56	10.8 ± 0.68	4.1 ± 0.28^a^	7.8 ± 0.5^b^	≤ 0.001*
MDA (nmol/g tissue)	51.7 ± 2.5	50.4 ± 2.9	91.4 ± 4.7^a^	65 ± 3.2^b^	≤ 0.001*
Catalase (U/g tissue)	19.6 ± 0.86	19.7 ± 0.78	8.6 ± 0.64^a^	14.2 ± 0.56^b^	≤ 0.001*
AChE (ng/ml)	4.1 ± 0.4	3.9 ± 0.2	13.5 ± 0.7^a^	6.9 ± 0.4^b^	≤ 0.001*
IL-1β (pg/ml)	3.7 ± 0.3	3.5 ± 0.2	8.2 ± 0.7^a^	5.04 ± 0.5^b^	≤ 0.001*
IL-6 (pg/ml)	28.6 ± 1.5	27.9 ± 1.1	58.9 ± 2.5^a^	36.6 ± 1.5^b^	≤ 0.001*

a Difference between the Sco and control groups (*p* value ≤ 0.001).

b Difference between the Sco + Rn and Sco groups (*p* value ≤ 0.001).*p value ≤ 0.05.

At the same time, rats in the Rn + Sco group showed a significant elevation in the levels of SOD, GSH, and catalase activity (*p* ≤ 0.001 for all) and a significant decrease in the levels of MDA and AChE activity (*p* ≤ 0.001 for both) as compared to rats in the Sco-treated group. However, no significant difference was detected between the control and Rn groups.

Furthermore, rats in the Sco group demonstrated a significant increase in the levels of IL-6 and IL-1 (*p* ≤ 0.001 for all) as compared to those in the control group, whereas, rats in the Rn + Sco group demonstrated a significant decrease in the levels of IL-6 and IL-1 (*p* ≤ 0.001 for all) as compared to those in the Sco group. On the contrary, no significant difference was detected between the control and Rn groups.

### 3.3 Effects of scopolamine and ranolazine on caspase-3 and BDNF gene expression in hippocampal tissue from the four study groups

As shown in Table 3, rats in the Sco-treated group demonstrated a significant increase in caspase-3 and a decrease in BDNF gene expression (*p* ≤ 0.001 for all) as compared to the control group, whereas, rats in the Rn + Sco group demonstrated a significant decrease in the gene expression of caspase-3 and an increase in BDNF gene expression (*p* ≤ 0.001 for all) as compared to those in the Sco group. On the contrary, no significant difference was detected between the control and Rn groups.

**Table 3:** Effects of scopolamine and ranolazine on caspase-3 and BDNF gene expression in the hippocampal tissue from the four study groups.

	Control	Rn	Sco	Rn + Sco	*p* value
Caspase-3	0.9 ± 0.08	0.9 ± 0.03	2.3 ± 0.2^a^	1.2 ± 0.09^b^	≤ 0.001*
BDNF	0.99 ± 0.04	0.98 ± 0.03	0.37 ± 0.05^a^	0.8 ± 0.05^b^	≤ 0.001*

a Difference between the Sco and control groups (*p* value ≤ 0.001).

b Difference between the Sco + Rn and Sco groups (*p* value ≤ 0.001).*p value ≤ 0.05.

### 3.4 Histopathological assessment of hippocampal tissue sections in the study groups

**Figure 1:**
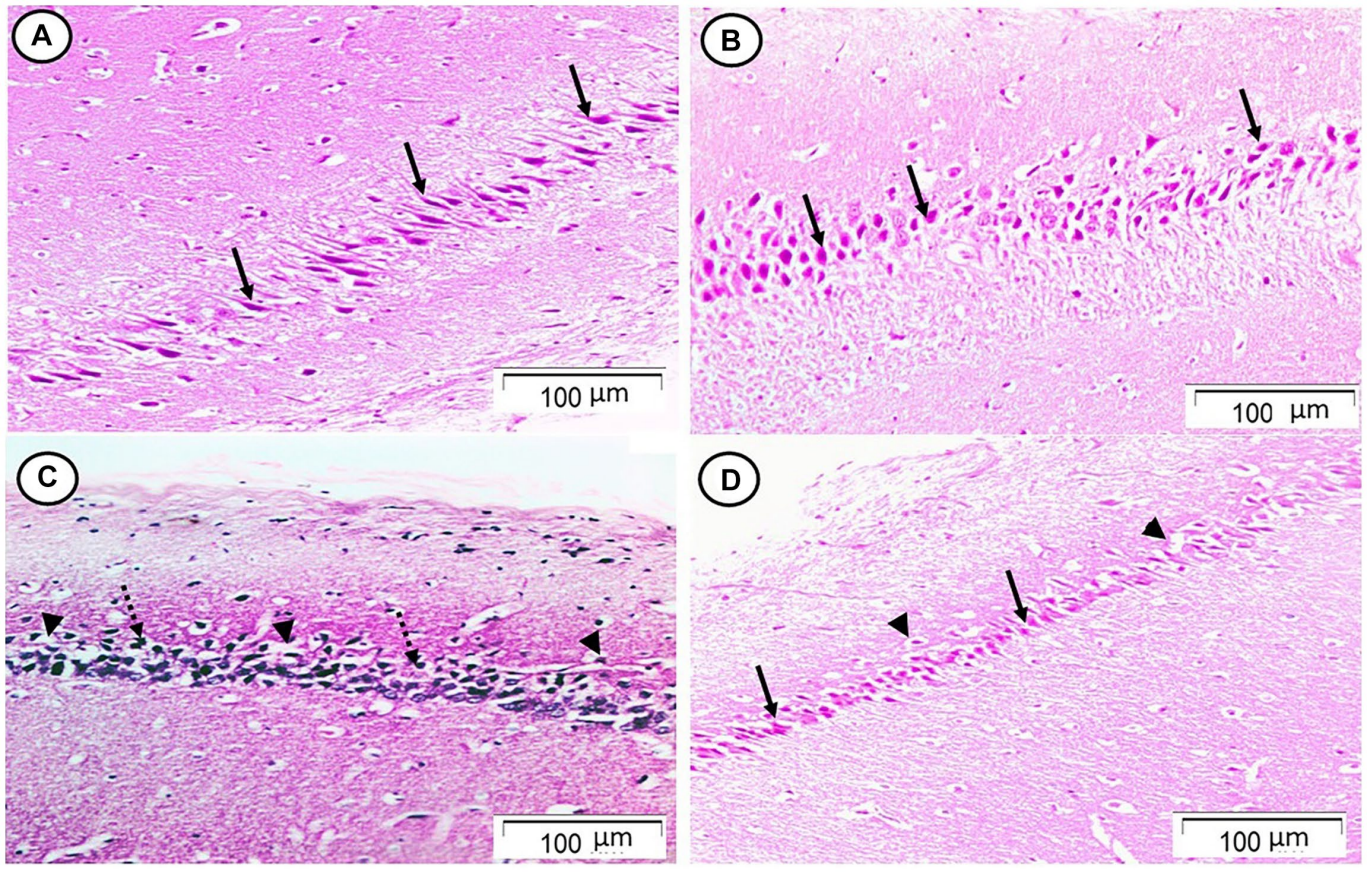
Photomicrograph of hippocampal cornu ammonis region 1 (CA1). **(A,B)** The control and Rn groups, respectively, showed the pyramidal cell layer formed of small pyramidal neurons with large vesicular nuclei and notable nucleoli (arrows). **(C)** The Sco group showed that most of the pyramidal neurons appeared to be deeply stained with pyknotic nuclei (dotted arrows). There were some halos that indicated neuronal loss (arrow heads). **(D)** The Rn + Sco group showed that most of the pyramidal neurons appeared normal (arrows). However, there were halos indicating neuronal loss still seen (arrow heads) (H & E, x 200).

Sections from the cornu ammonis region 1 (CA1) pyramidal cell layer of the Rn group revealed typical architecture as small pyramidal neurons with visible nucleoli and big vesicular nuclei, while those from the Sco group, however, demonstrated certain empty areas that suggested neuronal degeneration that could be seen among the majority of the pyramidal neurons, which looked to be strongly marked with pyknotic nuclei. Sections from the Rn + Sco group demonstrated that the majority of pyramidal neurons have big, vesicular nuclei, and visible nucleoli, giving them a generally normal appearance. However, there were hollows that suggested neuronal death (Figure 1).

### 3.5 Effects of ranolazine and scopolamine on the hippocampal tissue expression of tau protein in the study groups

**Figure 2:**
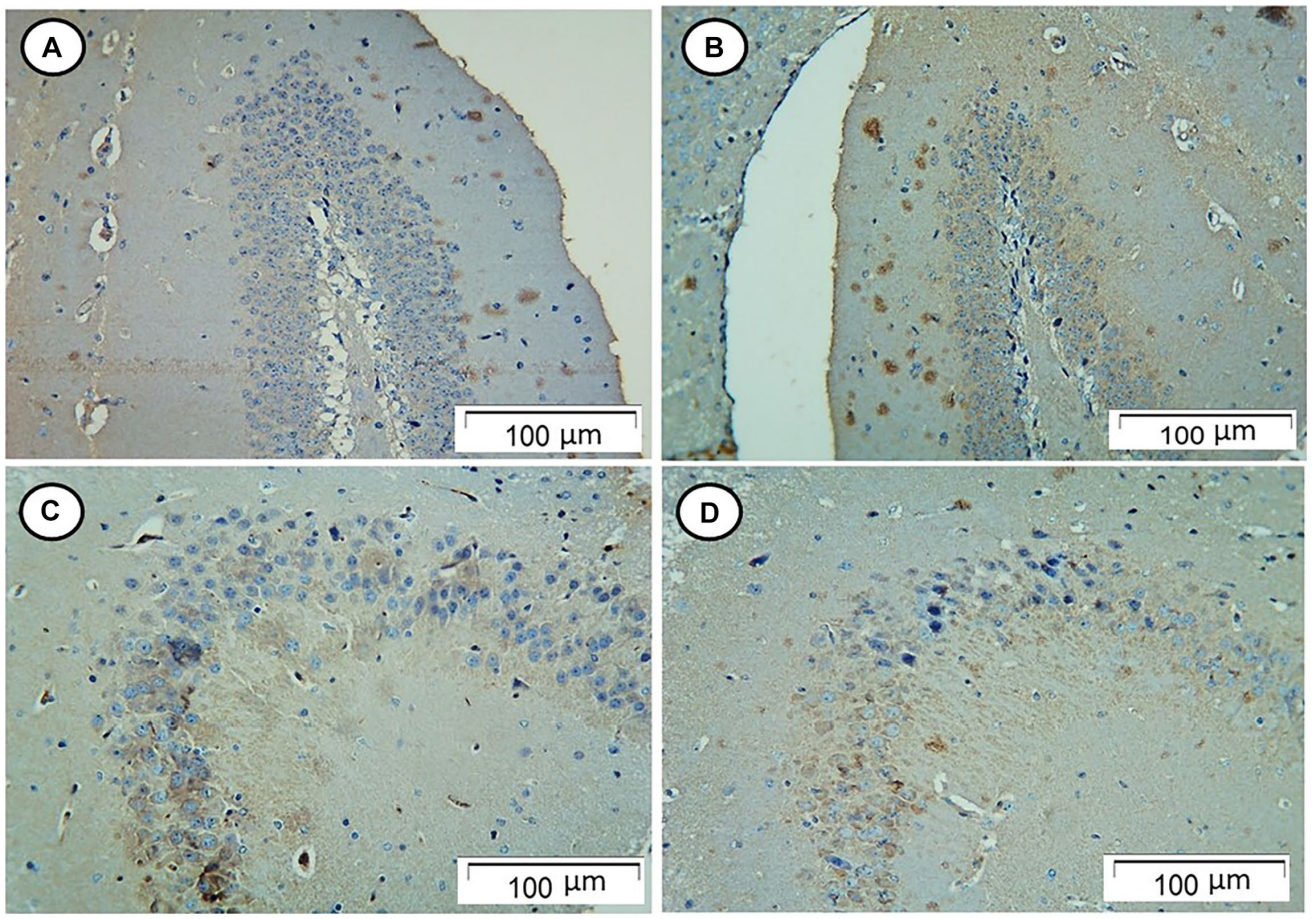
Photomicrograph of hippocampal sections. **(A,B)** The control and Rn-treated rats, respectively, showed negative tau immunostaining. **(C)** The Sco-treated group showed strong positive tau immunostaining. **(D)** The Rn + Sco group showed weak positive tau immunostaining (Tau, x 200).

The control and Rn-treated rats showed negative tau immunostaining. However, rats in the Sco-treated group showed strong positive tau immunostaining, while those in the Rn + Sco group showed weak positive tau immunostaining (Figure 2).

### 3.6 Effects of ranolazine and scopolamine on the hippocampal tissue expression of GFAP in the study groups

**Figure 3:**
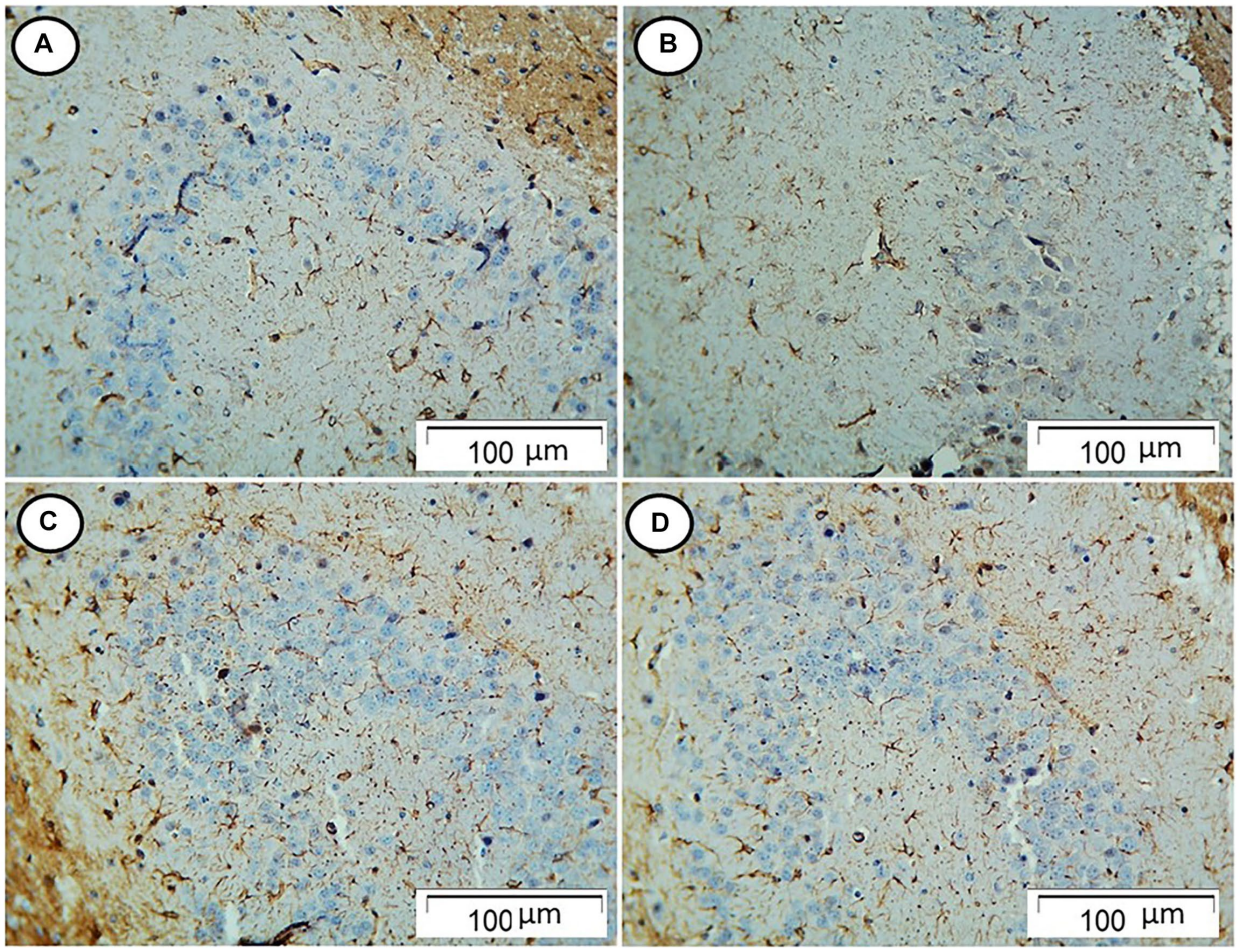
Photomicrograph of hippocampal sections. **(A,B)** The control and Rn-treated rats, respectively, showed few GFAP-positive cells. **(C)** The Sco group showed an increased number of GFAP-positive cells. **(D)** The Rn + Sco group showed a decreased number of GFAP-positive cells (GFAP, x 200).

The control and Rn group rats showed few GFAP-positive cells. On the contrary, rats in the Sco group showed an increased number of GFAP-positive cells. However, those in the Rn + Sco group showed a decreased number of GFAP-positive cells (Figure 3).

### 3.7 Effects of ranolazine and scopolamine on hippocampal tissue tau area percentage of positive reaction in the study groups

**Figure 4:**
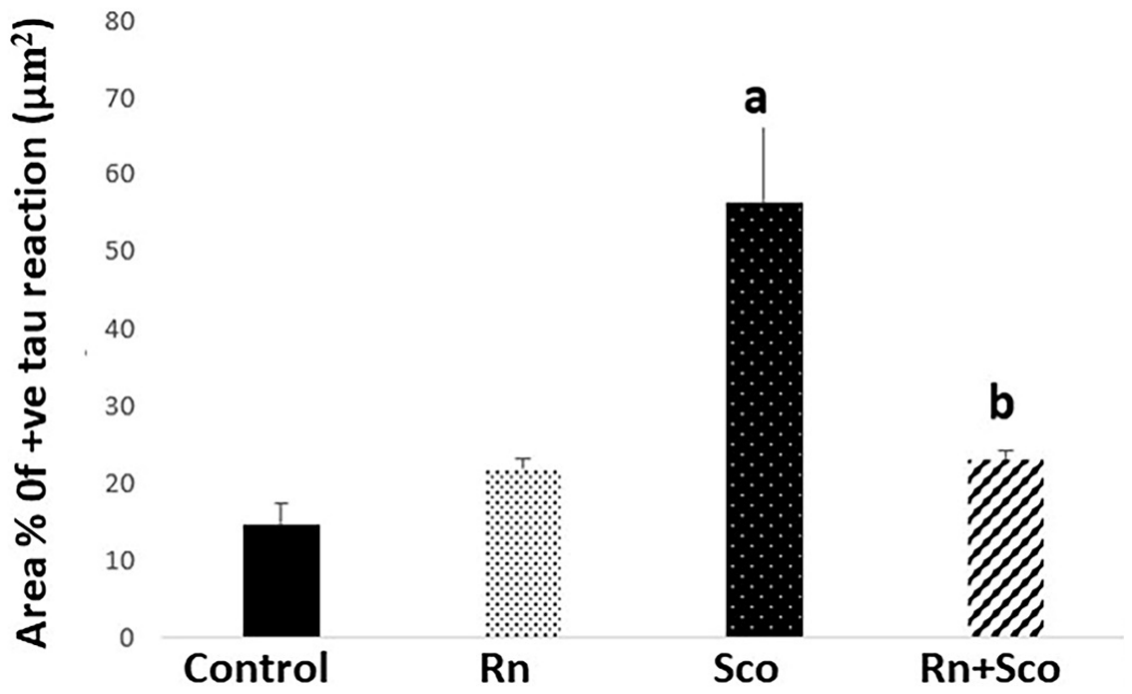
Area% of positive tau reaction. a: Difference between the Sco and control groups (*p* value ≤ 0.001). b: Difference between the Sco + Rn and Sco groups (*p* value ≤ 0.001).

As shown in Figure 4, rats in the Sco group exhibited a significant increase in the area% of positive tau immune reaction (*p* ≤ 0.001) as compared to the control group. At the same time, rats in the Rn + Sco group exhibited a significant decrease in the area% of positive tau immune reaction (*p* ≤ 0.001) as compared to those in the Sco group. However, no significant difference was detected between the control and Rn groups.

### 3.8 Effects of ranolazine and scopolamine on hippocampal tissue GFAP area percentage of positive reaction in the study groups

**Figure 5:**
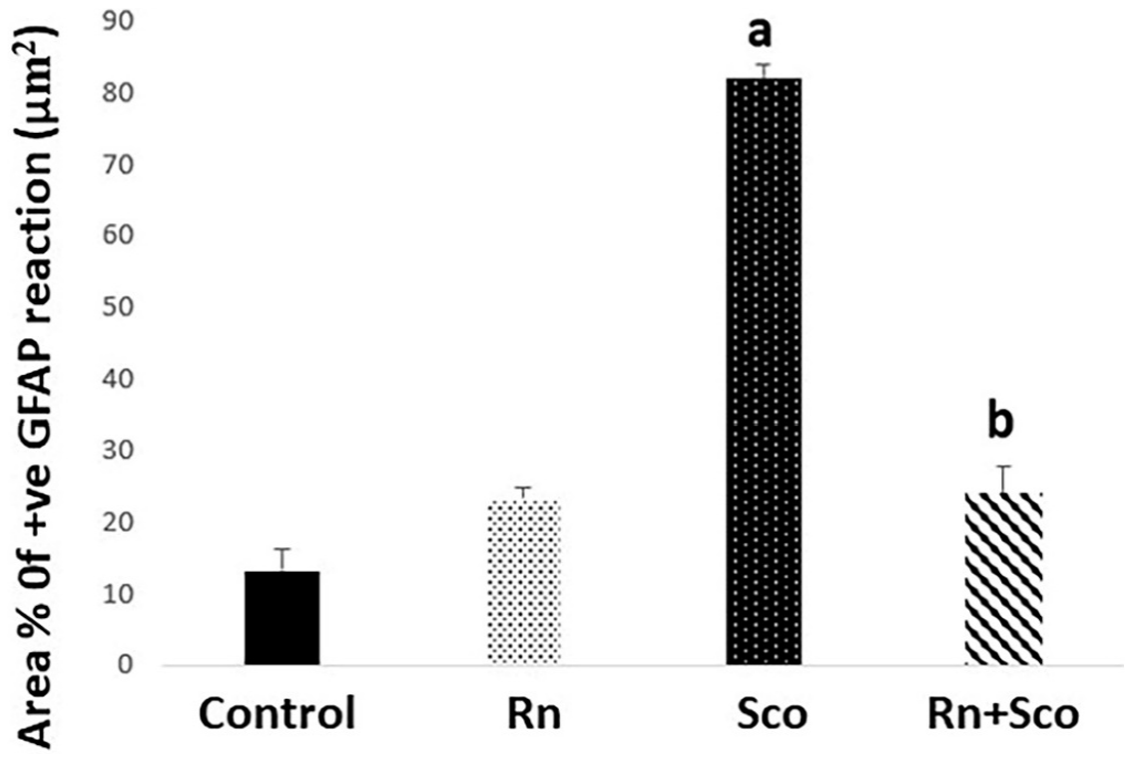
Area% of positive GFAP reaction. a: Difference between the Sco and control groups (*p* value ≤ 0.001). b: Difference between the Sco + Rn group and Sco groups (*p* value ≤ 0.001).

As shown in Figure 5, rats in the Sco group exhibited a significant increase in the area% of positive GFAP immune reaction (*p* ≤ 0.001) as compared to the control group. At the same time, rats in the Rn + Sco group exhibited a significant decrease in the area% of positive GFAP immune reaction (*p* ≤ 0.001) as compared to those in the Sco group. However, no significant difference was detected between the control and Rn groups.

## 4 Discussion

This study was designed to construct an experimental model of scopolamine-induced dementia in adult male rats and to evaluate the potential neuroprotective properties of ranolazine. The results obtained from the present study were of special concern as they provided clear evidence of the protective effect of ranolazine against memory deficits and cognitive impairment associated with scopolamine exposure.

Alzheimer’s disease (AD) is one of the most prevalent neurodegenerative disorders that is marked by the loss of memory, a reduction in cognitive abilities, abnormal behavior, and mental issues (Simunkova et al., 2019). Moreover, AD is the main cause of dementia affecting the elderly (Tang, 2019).

The Morris water maze test (MWM) was used in the present research to confirm the construction of the scopolamine (Sco) model of cognitive decline and memory impairments. The MWM test is a hippocampus-dependent memory test that is commonly used in rodents to show cognitive deficiencies in reference memory and permanent spatial learning ability (Doguc et al., 2012; Weitzner et al., 2015).

The present study found that Sco administration prolonged the time taken by rats to reach the platform (escape latency) compared with the control group. This result was in line with research by Park et al. (2016) and Hafez et al. (2017) that showed cognitive damage in spatial learning and memory function in rats following exposure to Sco.

However, the results of the current investigation showed that ranolazine (Rn) increased learning and memory as shown by a considerable reduction in the escape latency in the MWM test when compared to the rats treated with Sco. In agreement with this result, Cassano et al. (2022) showed that Rn therapy has protective benefits against the onset of cognitive deterioration in a model of type 2 diabetes in rats. In addition, Kahlig et al. (2014) concluded that the protective effects of Rn on the brain were due to decreased hippocampal neuronal excitability. Furthermore, Chunchai et al. (2022) demonstrated that Rn has neuroprotective benefits on the brain by restoring cognitive function and suppressing any brain diseases in rat models.

Oxidative stress is a significant factor in the development of AD (Cassidy et al., 2020). MDA is the product of lipid peroxidation (Zarrouk et al., 2020), and SOD, catalase, and GSH are considered to be important antioxidants that drive off oxidative stress and free radicles (Kong et al., 2019).

In the present study, treatment with Sco resulted in oxidative damage in the hippocampal tissue as evidenced by the elevation of MDA level. This result was also associated with the reduction of endogenous antioxidant mediators such as GSH, catalase, and SOD in rats in the Sco-treated group compared with those in the control group. Our findings concurred with those of Demirci et al. (2017) and Hosseini et al. (2022), who showed that Sco-induced dementia in rats was characterized by increased brain oxidative stress and lipid peroxidation, as well as decreased brain antioxidant reserves.

In our research, pre-treatment with Rn considerably reduced the oxidative stress caused by Sco by a decrease in the MDA level and an increase in antioxidant markers (GSH, catalase, and SOD). According to Chunchai et al. (2022) findings, Rn exerted its neuroprotective effects by reducing brain apoptosis, ameliorating brain mitochondrial dysfunction, enhancing blood brain barrier (BBB) integrity, and reducing brain inflammation, which led to an improvement in cognitive abilities. Additionally, Dogan et al. (2023) reached the conclusion that Rn′s antioxidant properties caused cardiomyocyte cells treated with Rn to exhibit lower levels of oxidative stress markers and higher levels of antioxidant capacity markers.

One of the principal neurotransmission routes in the brain involved in memory and cognitive processing is the central cholinergic system. The first pathophysiological element of AD was the considerable decrease in cholinergic activity (Blake et al., 2014). A change in the synthesis of acetylcholine comes from the neurodegeneration of cholinergic neurons and leads to the progressive deterioration of memory function (Hafez et al., 2017).

The current study’s findings demonstrated that Sco enhanced AchE enzyme activity in the hippocampus tissue of treated rats. According to Liu et al. (2020), Mostafa et al. (2021), Park et al. (2016), and Venkatesan (2022), Sco caused a significant deficit in cholinergic neurons and enhanced AChE activity and expression in the hippocampus, which exacerbated neurodegeneration in the brain. The current data support these findings. Additionally, Palle and Neerati (2017) excluded the fact that inhibition of the AchE enzyme leads to the presence of sufficient acetylcholine requirements for proper cognition, learning abilities, and memory retention in the brain. Additionally, it has been noted that Sco inhibits working memory-related muscarinic cholinergic receptors (Pezze et al., 2017).

Our research, on the other hand, revealed that the positive effects of Rn on cognition and memory might be triggered by a decrease in AChE activity and an increase in Ach levels in the hippocampus tissue. AChE has been shown to have a variety of non-classical, non-cholinergic roles in addition to metabolizing acetylcholine, including the processing and deposition of amyloid beta protein A (Aβ) (Castro and Martinez, 2006). Additionally, it was found by Zimmermann et al. (2005) that short-term AChE inhibitor therapy improves defective Aβ metabolism.

Another mechanism in the pathophysiology of AD is neuroinflammation. According to Wang et al. (2015), pro-inflammatory cytokines produced by brain microglia, such as IL-1, IL-4, and tumor necrosis factor (TNF), have been linked to the advancement of AD.

In our study, rats given Sco treatment exhibited greater levels of pro-inflammatory cytokines in the hippocampus, such as IL-1 and IL-6. This outcome was consistent with the findings of Demirci et al. (2017) and Shabani and Mirshekar (2018), who showed that rats exposed to Sco had hippocampus tissues with higher levels of pro-inflammatory cytokines. However, pre-treatment with Rn alleviated the inflammatory effect of Sco as indicated by the low levels of IL-1 and IL-6 in the hippocampal tissue. This outcome was verified by Chunchai et al. (2022) findings that Rn has an anti-inflammatory effect on the heart and brain. Rn was also reported to reduce TNF-α and IL-1 expression in cultured astrocytes and the sciatic nerve of diabetic rats (Aldasoro et al., 2016; Elkholy et al., 2020).

On the other hand, neuronal survival and neurogenesis in CNS are dependent upon neurotrophins such as BDNF, which is known to play a vital role in neuronal cell survival and the prevention of neurodegeneration; in addition, it is a central driver of synaptic plasticity for learning and memory (Numakawa and Odaka, 2022).

Studies have shown that BDNF plays a crucial function in memory and learning. It is significantly expressed in the cortical and hippocampal regions. Age-related changes in BDNF have been suggested as a potential cause of cognitive deterioration (Numakawa and Odaka, 2022). Moreover, BDNF expression is regulated by a transcription factor cAMP-response element binding protein (CREB) through its phosphorylation by various pathways (Li et al., 2015). CREB is necessary for memory and synaptic plasticity in CNS (Ortega-Martínez, 2015), and the disturbance of its phosphorylation leads to progressive neurodegeneration in the hippocampus (Mantamadiotis et al., 2002). As a result, the development of cognitive impairments and a reduction in CREB and BDNF production in the hippocampus are closely related (Li et al., 2015).

According to earlier research, we discovered that rats given Sco treatment had a notable reduction in BDNF gene expression in the hippocampal tissue. Our findings were consistent with those of Mansouri et al. (2021), who reported that the Sco injection impaired learning and memory by lowering BDNF levels. On the other hand, pre-treatment with Rn increased the expression of the BDNF gene, which may account for the protective effects of Rn on hippocampus neuronal cells.

Inadequate apoptosis has been linked to neurodegenerative diseases such as AD. Learning and memory are negatively impacted by the hippocampus and cortical neuron apoptosis. Caspase-3 is crucial for both inflammation and apoptosis (Tang, 2019).

Moving to our results, we found enhancement of apoptosis in the hippocampal tissue of Sco-treated rats, as indicated by the increased expression of the caspase-3 gene. Studies by Afzal et al. (2022) and Venkatesan et al. (2016) were in accordance with our results, as they found that scopolamine commenced apoptosis by the activation of caspase-3-directed apoptotic cell death with subsequent neurodegeneration. Rn additionally suppressed apoptosis by reducing the hippocampal tissue’s expression of the caspase-3 gene. Multiple studies have found reduced caspase-3 activity after Rn therapy in primary astrocyte culture cells (Aldasoro et al., 2016), rat atrial tissue (Zou et al., 2016), and rat cardiomyocytes (Chen et al., 2020), all of which are consistent with our finding.

Moreover, histopathological examination of the hippocampal tissue of Sco-treated rats revealed neuronal degeneration as indicated by deep staining of pyramidal neurons with pyknotic nuclei. These results were in agreement with Olayinka et al. (2022), who demonstrated Sco-induced cell degeneration and death in the hippocampus and the prefrontal cortex of rats.

However, Rn pre-treatment demonstrated neuroprotective effects since the majority of pyramidal neurons had large vesicular nuclei and prominent nucleoli, appearing more or less normal. These findings are supported by the findings of Aldasoro et al. (2016), who hypothesized that by increasing astrocyte viability, reducing necrosis and apoptosis, regulating inflammatory processes, and activating anti-inflammatory and antioxidant agents in primary culture cells, Rn may function as a neuroprotective medication in the CNS. Additionally, Chunchai et al. (2022) demonstrated that Rn protected the brain by reducing gliosis, apoptosis, and dysplasticity.

Hyperphosphorylated tau protein is one of the neuropathological indicators of AD (Jagust, 2018). According to Liu et al. (2015), tau hyperphosphorylation and oxidative stress have a close relationship. Therefore, ranolazine’s antioxidant activity, which was demonstrated in this study, may be related to its neuroprotective properties.

Immunohistochemical findings of the current study showed that the Sco-treated group had a strong positive tau immune reaction in the hippocampal tissue while pre-treatment with Rn showed weak positive immune reaction. These results were in accordance with the studies of Hafez et al. (2017) and Mandour et al. (2021) who found overexpression of p-tau protein in the brain tissue of Sco-treated rats. Regarding ranolazine, Chunchai et al. (2022) found that AD-related proteins such as tau remained high in ranolazine-treated doxorubicin rats.

The inflammatory response in the brain is characterized by the activation of glial cells and astrocytes. The expression of GFAP is considered a marker for this activation (Cassano et al., 2022). In our study, Sco induced the increased proliferation of reactive astrocytes in the hippocampus, which was indicated by an increased area% of GFAP-positive reaction. In contrast, the Rn pre-treated group showed the reverse. These findings were in accordance with those of Memudu and Adewumi (2021), who found an increased level of neuroinflammation biomarker GFAP in the cerebello-hippocampal cortex of Sco-treated rats. In addition, Cassano et al. (2022) demonstrated a decreased level of GFAP in the brain tissue of the type 2 DM rat model.

The results of the current research revealed that Sco-induced dementia in rats may be prevented by Rn administration. This outcome might be explained by Rn′s capacity to inhibit oxidative stress, apoptosis, inflammation, tau hyperphosphorylation, astrocyte proliferation, and overactivity of the AChE enzyme. As a result, the current study demonstrated that Rn could be a candidate to be used in the dementia regimen. However, to support the findings of our experiment, clinical trials are suggested.

## 5 Conclusion

The results of the current research exhibited that scopolamine-induced dementia in rats may be prevented by ranolazine. This outcome might be explained by ranolazine’s capacity to inhibit oxidative stress, apoptosis, inflammation, and astrocyte proliferation. As a result, the current study demonstrated that ranolazine is a candidate to be used in the dementia regimen. However, to support the findings of our experiment, clinical trials are suggested (Graphical abstract).

## 6 Limitations of the study

To wholly realize how ranolazine ameliorated dementia hippocampal changes in various experimental circumstances, more research is required.

## Data availability statement

The datasets presented in this study can be found in online repositories. The names of the repository/repositories and accession number(s) can be found in the article/supplementary material.

## Ethics statement

The animal study was approved by Mansoura University-Animal Care and Use Committee (MU-ACUC) (code number: MED.R.23.06.17). The study was conducted in accordance with the local legislation and institutional requirements.

## Author contributions

SS: Conceptualization, Methodology, Writing – original draft, Writing – review & editing, Investigation, Visualization. HH: Conceptualization, Methodology, Writing – original draft, Writing – review & editing, Data curation, Formal analysis. RE: Investigation, Methodology, Writing – original draft, Writing – review & editing. EE: Funding acquisition, Resources, Writing – review & editing. MaA: Funding acquisition, Resources, Writing – review & editing. MoA: Resources, Writing – review & editing. NA-Z: Funding acquisition, Resources, Writing – review & editing. FA: Resources, Writing – review & editing. ME: Conceptualization, Methodology, Writing – original draft, Writing – review & editing.
